# Assessing the Feasibility of Replacing Subjective Questionnaire-Based Sleep Measurement with an Objective Approach Using a Smartwatch

**DOI:** 10.3390/s23136145

**Published:** 2023-07-04

**Authors:** Maksym Gaiduk, Ralf Seepold, Natividad Martínez Madrid, Juan Antonio Ortega

**Affiliations:** 1 Department of Computer Science, HTWG Konstanz—University of Applied Sciences, 78462 Konstanz, Germany; 2 School of Informatics, Reutlingen University, 72762 Reutlingen, Germany; 3 School of Computer Engineering, University of Seville, 41012 Seville, Spain

**Keywords:** objective and subjective sleep measurement, sleep diary, sleep study, smartwatch, wearables

## Abstract

In order to ensure sufficient recovery of the human body and brain, healthy sleep is indispensable. For this purpose, appropriate therapy should be initiated at an early stage in the case of sleep disorders. For some sleep disorders (e.g., insomnia), a sleep diary is essential for diagnosis and therapy monitoring. However, subjective measurement with a sleep diary has several disadvantages, requiring regular action from the user and leading to decreased comfort and potential data loss. To automate sleep monitoring and increase user comfort, one could consider replacing a sleep diary with an automatic measurement, such as a smartwatch, which would not disturb sleep. To obtain accurate results on the evaluation of the possibility of such a replacement, a field study was conducted with a total of 166 overnight recordings, followed by an analysis of the results. In this evaluation, objective sleep measurement with a Samsung Galaxy Watch 4 was compared to a subjective approach with a sleep diary, which is a standard method in sleep medicine. The focus was on comparing four relevant sleep characteristics: falling asleep time, waking up time, total sleep time (TST), and sleep efficiency (SE). After evaluating the results, it was concluded that a smartwatch could replace subjective measurement to determine falling asleep and waking up time, considering some level of inaccuracy. In the case of SE, substitution was also proved to be possible. However, some individual recordings showed a higher discrepancy in results between the two approaches. For its part, the evaluation of the TST measurement currently does not allow us to recommend substituting the measurement method for this sleep parameter. The appropriateness of replacing sleep diary measurement with a smartwatch depends on the acceptable levels of discrepancy. We propose four levels of similarity of results, defining ranges of absolute differences between objective and subjective measurements. By considering the values in the provided table and knowing the required accuracy, it is possible to determine the suitability of substitution in each individual case. The introduction of a “similarity level” parameter increases the adaptability and reusability of study findings in individual practical cases.

## 1. Introduction

It is well known that sleep is an essential part of our lives, and it has a significant influence on our well-being and also on our health, which has been confirmed in numerous scientific studies [1,2]. Therefore, it is of great importance to ensure healthy sleep [3]. This means that both the duration and quality should be kept at a sufficient level. This would be important in recovering mental, brain, and body states, among other things [4].

In case a person gets sleep problems, it is crucial to identify the cause and then apply the appropriate treatment promptly. According to one of the classifications, the methods for analysing sleep with the aim of identifying possible sleep disorders can generally be divided into two groups: objective and subjective measurement [5]. In subjective measurement, the values perceived by the person are measured [6,7]. Questionnaires or sleep diaries are often used for this purpose [8]. In turn, objective measurement is based on electronic devices that measure physiological signals from the human body [9]. These signals are subsequently analysed. A classic example of an objective approach and, at the same time, the standard for sleep analysis is polysomnography (PSG), which is very accurate yet time-consuming and costly [10].

There are advantages and disadvantages to both of these approaches. Furthermore, it is important to note that both approaches have limitations and can be influenced by subjective factors. The crucial fact is that they are typically used to detect different sleep disorders. For example, objective measurement (e.g., PSG or polygraphy) is often used to diagnose sleep apnoea syndrome or for sleep stage scoring [11,12]. When detecting other sleep disorders, such as insomnia, a subjective measurement is often performed and further analysed [13]. One of the most commonly used methods is a sleep diary. It can be used to monitor and adjust therapy according to changes, for example, in cognitive behavioural therapy for insomnia (CBT-I) [14].

Subjective measurement requires daily action by the user due to the need to fill in a questionnaire manually. This may reduce the number of days on which the data are collected, which has been confirmed in other studies [15]. For this reason, it could be beneficial if the required data collection could be automated through an electronic device while maintaining the accuracy of a questionnaire. Ultimately, it is a standard approach with years of experience and numerous collected data sets to diagnose some sleep disorders, such as insomnia. Therefore, it would be vital to continue to have similar accuracy and validity.

Several research studies have addressed the question of replacing subjective measurement in sleep medicine with objective measurement or vice versa, including wrist-worn devices and an electronic sleep diary [16,17]. Some have used a device placed on the bed and providing a high level of comfort to evaluate the measurement results [18]. Others have undertaken other types of devices that allow a comfortable and exact measurement such as wearables as the device to be compared [19,20]. With such a variety of different devices, it is necessary to perform an evaluation of each type in order to obtain reliable results due to different measurement approaches. While in [15] the device placed under the mattress was evaluated, the focus of this work is on a category of wearable devices. Furthermore, various devices have different types of sensors on the board, are placed differently, and, moreover, development is constantly producing new devices with new features and characteristics. Therefore, generalising the results cannot give an accurate answer as to whether a particular device could be used for an adequate substitution of subjective measurement in a specific area of sleep medicine. Additionally, the possibility of substitution depends on the type of subjective measurement, which means that the validity of the measurement made with the objective method depends on the questionnaire to be substituted. Consequently, it is not a generalisation but an individual assessment of the devices and their field of application that should enable precise results. The scientific reviews carried out in previous studies have also indicated that further research is needed to provide reliable and accurate results on the possibility of substituting one type of measurement for another [21,22].

This work’s main objective is to evaluate the possibility of measuring relevant sleep-related parameters with a particular smartwatch (Samsung Galaxy Watch 4) as a sensor-based approach instead of a sleep diary in a real-life setting. This could lead to the solution of the problem with user comfort through the automation of the measurement process and ensure high data availability by excluding the possibility of forgetting to complete the questionnaire on time.

## 2. Materials and Methods

The specified research objectives were analysed in order to define the appropriate methods for their achievement. A detailed description of the selected methods is presented in the current section.

### 2.1. Subjective Sleep Diary-Based Measurement

When measuring sleep using a questionnaire, individuals may subjectively perceive their sleep quality differently due to factors such as stress, mood, or cognitive biases. Nevertheless, in the practice of sleep medicine, a sleep diary is one of the most commonly used methods for subjective assessment of sleep [23]. The version used to conduct this study is the German version recommended by the German Society for Sleep Research and Sleep Medicine (DGSM) because this study was conducted in Germany and this version has already been evaluated and used in several trials [24]. A total of 18 items are asked in this sleep diary, some of which consist of several questions. The questionnaires should be filled out daily in the evening and in the morning. Some examples of the questions included and relevant for the parameters to be measured within the conducted study are according to [23]:

- At what time did you go to bed?
- At what time did you try to go to sleep?
- How long did it take you to fall asleep?
- How many times did you wake up, not counting the final wake-up?
- How long did these awakenings last in total?
- At what time did you finally wake up?
- What time did you get out of bed?
- How would you rate the quality of your sleep?

In addition to the information extracted directly from the questionnaires, several additional sleep-relevant parameters can be determined by assessing the answers. Some examples are total sleep time (TST), total time in bed (TIB), and sleep efficiency (SE). The results of the sleep diary evaluation play an essential role in diagnosing and monitoring the evolution of some sleep disorders, e.g., in the case of insomnia [25].

### 2.2. Objective Sensor-Based Measurement

Various devices can be used in the objective measurement of sleep parameters. The accuracy of sleep stage identification, as well as other sleep-related characteristics, depends on the method of signal acquisition and can also vary between different algorithms applied to the captured signals, introducing a degree of subjectivity into the interpretation of sleep data. In addition, individual variations in sleep patterns and characteristics may affect the reliability of smartwatch sleep estimations in individual cases.

If the goal is to enable comfortable use for users, it is desirable that the device does not disturb the user during sleep or disturb him as little as possible. To achieve this, there are different approaches as to which devices can be used and placed [26]. One viable option is a device placed under the mattress [27]. In this case, direct contact with the human body is not necessary, leading to a higher level of comfort. At the same time, accurate measurement is a challenging task due to the absence of a direct connection of a sensor to the human body. If direct contact with the body is not an exclusion criterion, another commonly used approach can find the application: the use of wearables [28]. Such devices can be comfortably worn on the human body. The category of wearables consists of several types of devices [29], one of which is a smartwatch facilitating comfortable yet accurate measurement [30].

An analysis was carried out to enable the selection of the appropriate device. Preliminary usability testing and research on existing scientifically conducted evaluations were performed for the following devices: Fitbit Sense, Samsung Galaxy Watch 4, Amazfit GTR 3 Pro, and Huawei Watch 3 Pro. Ultimately, a decision was made to consider the Samsung Galaxy Watch 4 for several reasons. The evaluation of previous models of the Samsung Watch that have assessed the measurement of physiological parameters with acceptable results has already been performed [31]. The sleep monitoring application [32] has also already been positively evaluated. Therefore, it can be assumed that the latest available version of the smartwatch would provide at least similar or even better results due to the use of the latest sensors and software system. This device can be used to measure the sleep parameters relevant to the study, such as the times of falling asleep and waking up, sleep efficiency, and sleep profile. The measurement results are either directly available in the corresponding mobile app or can be downloaded and analysed as part of the stored private data via the menu option in the mobile app. To set up the smartwatch, the mobile app can be used, which can be performed before the start of the study. This means that no further action is required before bedtime, increasing the convenience of use and minimising the number of potential problems during the study. The watch can be worn on any wrist during the day as well as at night, and the battery life is between 30 and 40 h, which is sufficient for the objectives of the planned study.

### 2.3. Sleep Characteristics

Subjective sleep measurement aims to record and analyse relevant parameters. They are either taken directly from a questionnaire/sleep diary or calculated from the filled-in points. The number of possible parameters is considerable; therefore, establishing a selection for the evaluation was necessary. One of the prerequisites was to evaluate the parameters relevant for insomnia diagnosis and therapy observation [33]. On the basis of the analysis carried out, several relevant parameters were selected for the evaluation of the possibility of replacing the sleep diary with an objective measurement:

- Falling asleep time
- Waking up time
- Total sleep time (TST)
- Sleep efficiency (SE)

These parameters can be measured directly as a part of the objective measurement with a smartwatch or calculated from the collected data. At the same time, they can be measured by answering the questions in a sleep diary, as described in the Section 2.1. Thus, the set of sleep characteristics used for the first evaluation was determined.

Since according to [34], there are different variants to calculate SE, and in order to eliminate possible misunderstandings in comprehending this article, we provide the formula used for its determination. The SE for subjective measurement was calculated in the study using the following Formula (1):

SE = TST/((Waking up Time − Time trying go to Sleep)) × 100% (1)

For the evaluation of the possibility of replacing subjective measurement with an objective one, the differences in measurement results between these two types of measurement must be calculated and analysed for every selected sleep characteristic.

### 2.4. Test Subjects

The list of inclusion and exclusion criteria was developed during the preparation stage based on the performed analysis of the objectives. The following *inclusion criteria* were established:

- Age: 20–60 years old (including adults without elderly due to possible differences in sleep patterns)
- No significant sleep disorders are known. (To decrease the chance of disorders affecting the results, participants were asked to provide this information and no additional screening was performed)
- Declaration of consent to participate in the study after explaining the study process and objectives (to follow ethical and legal requirements).

*Exclusion criteria* defined for the study:

- Chronic or acute clinically significant diseases occurring during the study. (No diagnostic tests were carried out in the study)
- Drug/alcohol abuse
- Significant perceived impairment of sleep due to the wear of smartwatches.

The sample size of 100 suggested by Israel [35] for a precision level of 10% with a confidence level of 95% and *p* = 0.5 was used as the basis for the definition of the number of recordings required. In order to increase the explanatory power, this number was increased according to the aim of collecting at least 150 recordings, having several nights pro participant to compensate possible outliers in single recordings caused by abnormal daily routine or some exceptional night occurrences. For this purpose, the recruitment of subjects for the study was started at the HTWG Konstanz in Germany, trying to contact a similar number of male and female persons as well as considering different age groups within the determined range. For this step, 15 subjects were contacted by the time this article was prepared. A total of 13 of them expressed their willingness to participate in the study. Next, the inclusion/exclusion criteria were applied, and two individuals had to be excluded due to the presence of insomnia in one of them and significant perceived sleep disturbance due to wearing the smartwatch in another (feeling of discomfort and the perception of sleeping worse than before), which only became apparent after the first few days of the experiment. After applying the inclusion/exclusion criteria, the group of subjects consisted of eleven people with an average age of 32.7 years (*SD* = 10.1, median = 32, range: 25 to 59 years) was available for the evaluation. The BMI of the participants was in the range of 20.5 to 29.9, with an average of 23.8 and median of 23.4. The number of male and female participants was similar (one more male participant) to facilitate a sex-independent analysis. During the study, participants were asked to maintain their usual lifestyle, and they remained in their homes for the execution of the experiment.

Only healthy adult volunteers participated in the study. Furthermore, the subjects were not patients, children, or elderly people with no diagnostic purpose and no clinical trial was conducted. In addition, it is essential to mention that the focus of this study was to assess the feasibility of replacing subjective sleep measurement with an objective approach using a publicly available consumer smartwatch and not a medical device. A study description (including information on aims, procedures, risks, etc.) and an informed consent and publication forms were first prepared to provide potential participants with this information in advance and to give them sufficient time to decide whether to participate and to answer any questions they may have. It is important to note that none of the physiological signals, such as heart or respiratory signals, were directly processed by the study organisers and will under no circumstances be made publicly available or shared with third parties. In addition, subjects were given ID numbers and all data were completely anonymised so that ID and subject could not be matched as there is no key. Age and BMI data were collected separately and were not linked to IDs or names to ensure a high level of anonymisation.

### 2.5. Experiment Design

A research team determined the design of the experiment conducted for the evaluation and was then followed by the study participants. The experimental procedure was introduced to the subjects on the first day, and smartwatches and sleep diaries were presented and explained. All the participants’ questions were addressed, and the devices were tried in use. Furthermore, all subjects gave their informed consent to be included in the study and publication of the results in an anonymised form prior to participation.

The regular daily routine of the test persons should be continued in order to be able to carry out a real-life evaluation. The aim was to examine whether substituting the sleep diary with the smartwatch is practicable in a real-life use scenario. To obtain reliable data, test subjects were asked to wear the smartwatches during the day and night, or at least to put them on 20 min before going to bed to ensure correct measurement. They were not allowed to take them off earlier than 20 min after getting up to facilitate automatic scoring by the smartwatch software. The smartwatches had to be recharged whenever the battery level was below 35% to ensure their functionality throughout the night. If there were any issues with the device, the subjects had to contact the study organisers and discontinue using it until resolved. Each evening, participants were asked to complete the evening part of a sleep diary immediately after going to bed. Directly after getting up in the morning, they were requested to fill in another part of a sleep diary with follow-up questions. The duration of the experiment consisted of 17 nights per participant; however, 3 participants had only 16 nights of recording duration due to their unavailability on the last day.

On the last day of the study, a discussion was held with the participants to obtain general feedback on the performed study. The sleep diaries were collected, and general (anonymised) information on age, height, and weight was gathered separately. The devices were also checked and picked up by the participants.

### 2.6. Statistical Analysis

Statistical analysis was performed to assess the feasibility of replacing subjective questionnaire-based sleep measurement with an objective approach using a smartwatch. Several statistical measures were used, including the Bland–Altman plot, the intraclass correlation coefficient (ICC), and the mean, median, and standard deviation (*SD*) of the differences between the two measurement methods.

To assess the agreement between the smartwatch and sleep diary measurements, a Bland–Altman plot was generated for each sleep characteristic [36]. The Bland–Altman plot is a graphical representation that can be used to examine the systematic bias and limits of agreement between two measurement methods. In this analysis, the differences between the smartwatch and sleep diary measurements were calculated for each participant and plotted on the y-axis, while the average of the two measurements was plotted on the x-axis. The graph includes a horizontal line representing the mean difference between the two methods and two additional lines, called limits of agreement, set at ±1.96 times the standard deviation of the differences. These limits represent a range within which approximately 95% of the differences between the two methods should fall if there is no systematic bias. 

The Bland–Altman plot provides valuable insight into the agreement and comparability of the smartwatch and sleep diary measurements. It helps to identify potential sources of discrepancy between the two methods and allows a more comprehensive assessment of their interchangeability. By using this statistical method, the study aims to gain a deeper understanding of the feasibility of replacing subjective questionnaire-based sleep measurement with the objective approach using a smartwatch.

In addition, the ICC was calculated using a two-way mixed effects model with absolute agreement and single rater/measurement [37]. The ICC is a statistical measure that assesses the proportion of the total variance in the measurements that can be attributed to true differences between subjects/methods. It is a measure of the consistency and agreement between the two methods, with values closer to 1 indicating greater reliability. As the result of a measurement should be a quantity that can be represented in a meaningful way by a real number, i.e., numerical data [38], the values for time of day (hh:mm) had to be converted to minutes (mm), and the calculation of the ICC for the SE, which is derived from time values and expressed as a percentage, and which naturally has a relatively high measurement variance with both methods, was not considered meaningful in the context of this study.

Furthermore, the mean, median, and SD of the differences between the smartwatch and sleep diary measurements were calculated for each sleep characteristic. These descriptive statistics provide an overview of the size and variability of the observed differences between the two measurement methods.

In addition, box plots were used to illustrate the differences between the smartwatch and sleep diary measurements for different sleep characteristics, including falling asleep time, waking up time, total sleep time, and sleep efficiency. A boxplot provides a concise visual summary of the data distribution and allows quick comparison between different measurement methods [39]. 

In each box plot, a rectangular box is drawn to represent the interquartile range (IQR) of the data. The bottom and top of the box represent the 25th and 75th percentiles, respectively, while the line inside the box represents the median. This median line is a measure of the typical difference between the two methods. The whiskers extend from the box to show the range of the data, excluding outliers. Outliers are shown as individual data points beyond the whiskers. These outliers may represent extreme differences between smartwatch and sleep diary measurements for some participants.

## 3. Results

The total number of recorded experiment nights was equal to 184. However, ten nights had to be excluded from the evaluation due to missing or obviously incorrect data in the sleep diary (for example, when the waking up time was reported to be later than the getting out of bedtime), and eight nights were excluded due to missing or incomplete data from a smartwatch (for example, when the smartwatch lost power during the night). The distribution of excluded recordings between study participants was wide; therefore, no significant effect was expected on the final results. Ultimately, 166 overnight recordings were available for evaluation and have been analysed. This number of collected data allows us to obtain statistically significant outcomes and consequently draw substantive conclusions.

The variation of the measured characteristics was also relatively high, as can be seen in Table 1, indicating that different sleep times were covered, which was important for a comprehensive analysis of the data due to the fact that sleep behaviour and consequently sleep times vary significantly among persons. Table 1 shows the statistical data on the measured values of each characteristic for all 166 recordings, both for the subjective sleep diary measurement and the objective measurement by means of the smartwatch.

For each parameter of interest, the absolute values of the differences between subjective and objective measurements were calculated for each of the 166 recording days. In the next step, statistical parameters, as well as visualisations for better comprehensibility of the evaluation results, were prepared as described in the Section 2.6.

**Figure 1:**
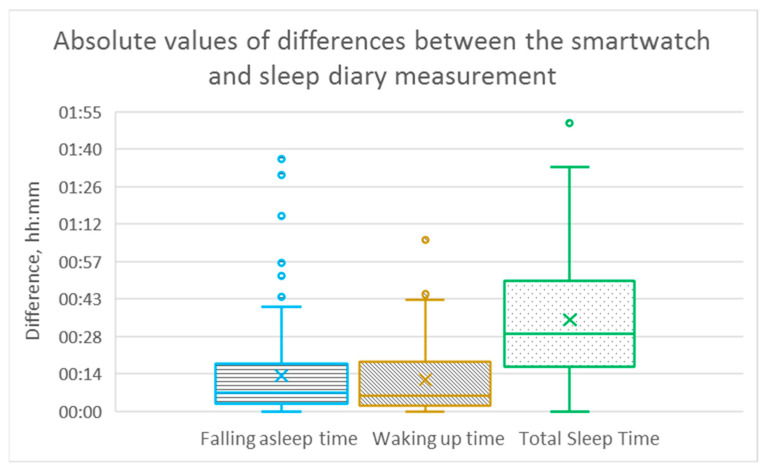
Box plots presenting the differences between the smartwatch and sleep diary measurement for “Falling asleep time”, “Waking up time”, and “Total Sleep Time” for 166 overnight recordings.

Figure 1 presents box plots with calculated differences between two measurement approaches for three sleep characteristics: falling asleep time, waking up time, and total sleep time. It can be observed that the number of outliers is significantly higher for the time of falling asleep compared to the time of waking up. The dispersion of differences between the two measurement methods for a time of falling asleep and waking up is meaningfully lower than for total sleep time.

**Figure 2:**
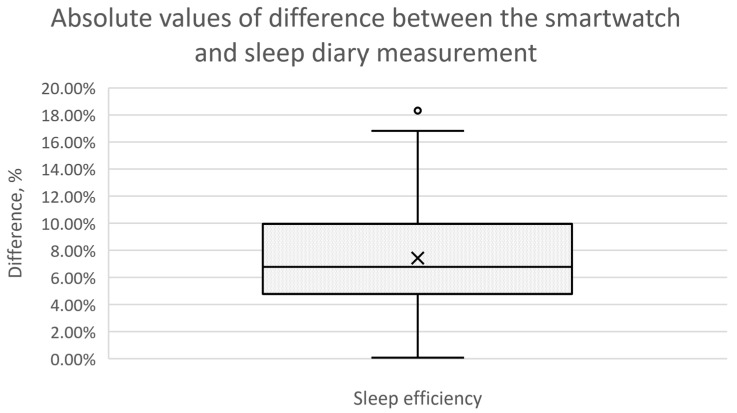
Box plot presenting the differences between the smartwatch and sleep diary measurement for the characteristic “Sleep Efficiency” for 166 overnight recordings.

For the fourth evaluated sleep characteristic, sleep efficiency, the visualisation can be seen in Figure 2. The mean and median values of the differences are very close for this characteristic, being noticeable in the graphical representation. This indicates that the dispersion of results is lower than for other measured characteristics.

The summary of the obtained within the statistical evaluation values for all four sleep characteristics is outlined in Table 2, providing a brief and coherent overview of the outcomes obtained as the result of the experiment carried out. The statistical values were selected according to the results of literature research on similar scientific studies with the aim of providing a meaningful overview.

**Figure 3:**
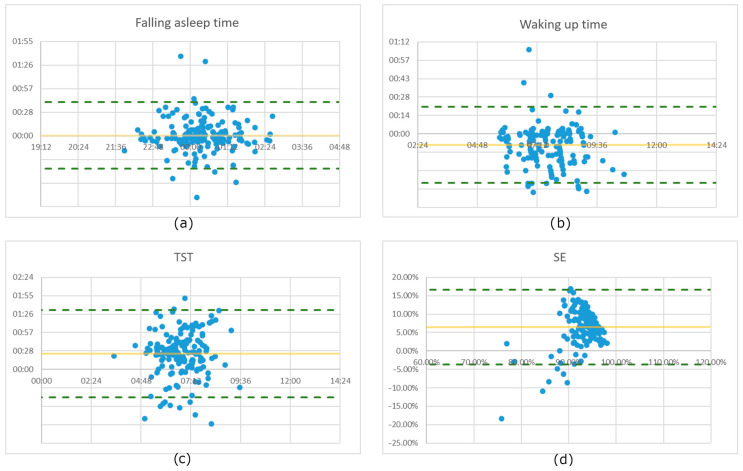
Bland–Altman plots for all analysed sleep characteristics for 166 nights: (**a**) Falling asleep time, (**b**) Waking up time, (**c**) Total sleep time, and (**d**) Sleep efficiency.

In order to facilitate a more in-depth analysis of the results obtained, we have prepared the graphical representation of the measurements using Bland–Altman plots for all four sleep characteristics, as shown in Figure 3.

The calculation of the ICC for “falling asleep time”, “waking up time”, and “TST”, both for each subject and for the 166 nights of recording, was performed after converting the time-of-day values (hh:mm) to minutes (mm) and gave the following results. For “falling asleep time”, the values range from 0.76 to 0.99 with the exception of one value of 0.68, along with a mean of 0.9, median of 0.93, *SD* = 0.096, and IQR = 0.057. The ICC for the sum of the 166 recordings is 0.93. For waking up time, the values range from 0.76 to 0.99 with a mean of 0.93, median of 0.95, *SD* = 0.075, and IQR = 0.086. For all 166 night recordings, the value is 0.96. This allows us to say that the ICC values for “falling asleep time” and “waking up time” indicate the reliability of the substitution of the sleep diary by the smartwatch measurement as “good” to “excellent” for individual subjects and “excellent” for the complete set of recordings according to [40]. For the TST, which includes several components, the ICC calculation yielded the following values: interval between 0.53 and 0.94, mean of 0.76, median of 0.75, *SD* = 0.115, and IQR = 0.115, while the ICC for 166 recordings for this characteristic is 0.76, indicating “good reliability”.

## 4. Discussion

The appropriateness of replacing the sleep diary measurement with a smartwatch depends on the acceptable level of deviation in the measurement results. As the level of acceptability can differ for individual cases (e.g., in some cases, a difference of 10% of SE is acceptable, while in other cases, no more than 5% of the difference is allowed), we provide four levels of “similarity” of the results for two measurement approaches. Each level defines a range of absolute differences between the objective and subjective measurements, and one of these can be selected depending on the specific requirement. This method of providing several levels of “similarity” has also been used in other publications, e.g., in [41], and can ensure a differentiated application of the results obtained, depending on the needs. For the characteristics “falling asleep time” and “waking up time”, the levels of 10, 20, 30, and 40 min of maximum difference between objective and subjective measurement methods were chosen. For TST (which is a characteristic that includes more components and therefore has a higher level of inaccuracy), the levels of 15, 30, 45, and 60 min were chosen. Finally, for SE, the values are 5%, 10%, 15%, and 20%. Table 3 shows the percentage of nights out of the 166 available study records for which the calculated difference between the sleep diary and the smartwatch measurement is within the corresponding “similarity” level. This means that, for example, if the table shows a value of 76.5% for the “time to fall asleep” parameter with “Similarity level 2” (20 min), we have a probability of 76.5% that the difference between subjective and objective approaches to measuring this parameter will be no more than 20 min.

Based on the values given in Table 3 and knowing the required level of accuracy, it can be decided whether or not replacing the subjective measurement with the objective method would meet the requirements in a particular case. Including this “similarity level” parameter significantly increases the added value of the work carried out, as its results can be adapted to the specific case of use, improving the “reusability” of the findings. 

When analysing the results obtained, one can see that the differences between the two measurement methods vary for diverse sleep characteristics. One of the reasons for the higher differences between subjective and objective measurement methods for the TST and SE parameters could be the fact that subjects typically significantly underestimate the time being awake during the night, i.e., wake after sleep onset (WASO), which was confirmed in other studies [42]. And this parameter is included in the calculation of TST and SE. Analysing in detail the available recordings has validated this assumption. The analysis of the study data did not reveal any very strong person-specific peculiarities, whereas mild differences were observed, particularly in those with a very regular sleep pattern, although the limited number of nights per person and thus the strong effect of outliers make it difficult to draw reliable conclusions on this point. Analysis of the Bland–Altman plots shows that for the vast majority of the 166 measurements, the differences are within the 95% confidence interval. Some exceptions can be explained by the fact that both methods sometimes give inaccurate results; in the case of the sleep diary measurement, the measurement is often inaccurate due to the subjective approach, and it cannot be guaranteed that subjects did not fill in the sleep diary in time on some days (i.e., not immediately after going to bed and getting up) and may not have known the exact times at the moment of filling it in. Even when measuring with a smartwatch, it is not always possible to get an absolutely accurate measurement, as both algorithmic and user errors are possible. In addition, if both measurement errors (sleep diary and smartwatch) are present on some of the measurement nights, the errors will be added together, which could lead to a higher total error value.

The previously listed differences between the two measurement methods and the calculated statistical parameters refer to absolute values. However, the “directions” of the typical discrepancies between objective and subjective measurement also represent vital information, i.e., it is meaningful whether persons tend to overestimate or underestimate some of the parameters compared to the measurement with the smartwatch. This analysis was also carried out in the context of this work, can be observed on the provided Bland–Altman plots and in Table 1, and the following can be noticed:

- For the time of falling asleep, the deviations between measurement methods are similarly widespread in both directions; in 88 of the 166 measurements, the objective measurement showed that the person fell asleep later than the subjective one.
- In the case of waking up time, there is a tendency that the time registered with the smartwatch is later compared to the sleep diary. This was the case for 114 nights. One of the reasons could be the fact that subjects keep lying in bed with no movement after waking up, which can be classified by smartwatch as “Sleep” time. This assumption was confirmed by a detailed analysis of the corresponding nights.
- The study showed that people often overestimated TST and SE when comparing the results with the objective measurement. This was observed in 136 recordings for TST and 152 for SE. This fact can be partially explained by the underestimation of the time being awake during the night, as discussed above, and it coincides with other studies [43,44].

Comparing the results of the performed study with other scientific works where subjective measurement was contrasted with an objective approach (however using not a smartwatch but different kinds of devices), we can see that a certain level of accordance in results can be observed. For example, in [45], the difference in TST measurement was about 40 min, having sleep diary and electroencephalography as measurement methods. The difference in SE measurement was, on average, approximately 7%, which is comparable to the results of our research. In [42], the difference of 85 min between the sleep diary and the TST actigraphy measurement was reported, which is significantly greater than the values obtained in our study values. The reason for this could be a more accurate measurement of sleep/wake states by Samsung Galaxy Watch 4 compared to actigraphy due to the use of several sensors measuring various characteristics and not only movement, as in the case of actigraphy. It confirms the statement made in the Section 1 that a comparison of objective and subjective measurement should be performed for specific devices and cannot be generalised to all objective measurement approaches.

## 5. Conclusions

Taking into account the fact that an automatic measurement of sleep characteristics would both increase comfort for the user and avoid potential data gaps by not requiring daily action on the part of the user, the evaluation of the possibility of replacing the sleep diary with an electronic device carried out in this study appears to be significant for future investigation and development of appropriate measurement systems. Different devices may use different types of sensors and software systems. A transfer of the results from one device to another or a generalisation of the results seems to be uncertain and may lead to a loss of appreciation of the particularities of the different devices. Therefore, it is necessary to evaluate a specific device in order to make an accurate statement about the possibility of the mentioned substitution.

For this study, the Samsung Galaxy Watch 4, one of the currently widespread smartwatches with up-to-date hardware and software components, was selected for evaluation. The conclusions presented below refer to this particular smartwatch model and are not intended to be directly generalised. As a summary of the results obtained within the scope of the study after their comprehensive analysis, the following statements can be outlined as the most important findings:

- The results correlate strongly with the subjective measurement when measuring the waking up time with an electronic device, which was also confirmed with the ICC calculation. Therefore, it can be concluded that the substitution of these two measurement methods for this parameter can be advisable.
- In the case of measuring the time of falling asleep, the results obtained with a smartwatch have a high accuracy when the subjective measurement is used as a reference as the performed analysis has demonstrated. This indicates the possibility of replacing the sleep diary measurement with the use of a smartwatch to measure the time of falling asleep.
- The evaluation of the measurement of TST as one of the vital sleep characteristics led us to the conclusion that the substitution of subjective measurement by objective measurement and vice versa is not always reliable when a high degree of accuracy is essential. One of the reasons for this could be the fact that in this characteristic both the inaccuracies in the measurement of the time of falling asleep and the time of waking up are summed up. Moreover, the phases of awakening during sleep are measured more clearly and longer by the device, while people tend not to perceive or underestimate these intervals.
- Another parameter evaluated—SE—seems to be suitable for replacing a sleep diary as a measurement tool with a Samsung Galaxy Watch considering a typical variation of this parameter and consequently some level of inaccuracy. The difference between the two measurement methods in the calculated value is, on average, about 7%, although the difference can be up to 18% in single cases. This leads us to the conclusion that the substitution can be inaccurate in some cases if only a single or a small number of nights are evaluated. For long-term observation, substitution appears to be more appropriate, especially if not absolute values, but the trend changes over time of interest, as the study performed has indicated that the difference between the two measurement approaches remains relatively stable over time.

The evaluation performed leads us to the conclusion that the substitution of a sleep diary measurement by measurement with a Samsung Galaxy Watch is possible for several relevant sleep characteristics if some level of inaccuracy is accepted. This may enhance the convenience of use and reduce a user’s time effort. As a further advantage, the automation of the process can reduce the amount of lacking data, which would positively affect the evaluation of the data, particularly when it comes to long-term observation. To allow one to make a well-founded decision if the substitution is acceptable in a particular case, four levels of imprecision for every characteristic were evaluated and summarised in Table 3, facilitating the possibility of deciding whether the substitution would provide a satisfactory outcome based on a known suitable level of inaccuracy.

However, it is important to remember that even when measuring with an electronic device, the results are not always completely objective, due to the measurement method, the measurement process, the algorithms used, etc. Despite the careful planning of the study and attempts to conduct a comprehensive evaluation, there are some limitations of the conducted study that should be mentioned:

- The evaluation is based on a dataset collected during the study, which includes 166 overnight recordings. This number should provide statistically significant results according to the established practice of conducting scientific research. Nevertheless, the transferability of the results to the entire population would be even more accurate if a larger sample was used.
- A specific device was included in the evaluation in order to obtain accurate data that could be replicated when using a device of the same type. As a result, it is not possible to generalise the results to all smartwatches due to the different hardware and software components used in different wearables.
- Although participants from different age groups were involved in the study, most (82%) were from the age group 25–35 years old, and consequently, the average age was relatively low. Furthermore, only adults have been included in the study. Accordingly, no precise statement can be made on whether the transferability of the results to all age groups is equally accurate.
- Due to the limited number of recordings per person and the correspondingly high influence of individual outliers (which are possible with both measurement approaches), it is not possible within the framework of the study to make a reliable statement about the degree of difference between the measurement methods for each individual and whether there are significant person-specific particularities.
- It is also not possible to make a statement about the transferability of the results to the group of people with (sleep) disorders, as the study was limited only to healthy people.
- Four sleep characteristics were the core subject of the research carried out. The results obtained in the context of the research conducted cannot be transferred to other sleep-relevant parameters and are limited to the evaluated parameters due to the unique characteristics of the measurement of different parameters and the varying accuracy depending on the characteristic.

When analysing the results of the conducted study and its evaluation, the conclusion can be drawn that, at the moment, no general recommendation can be made to replace sleep diary measurement with an objective approach using a smartwatch. At the same time, measuring some sleep-relevant parameters with a smartwatch instead of a sleep diary can currently be suggested to increase user convenience and reduce data loss if the acceptable level of discrepancy is known.

The results achieved already provide several insights and appear promising, but at the same time, some perspectives open up to overcome the listed study limitations and obtain results that are yet more comprehensive. Several directions have been established for future work:

- Additional studies with a larger number of participants and recording nights per subject are in the planning stage. Moreover, more subjects from different age groups will be included in the experiment, and the evaluation will be carried out both age group-specific and age group-across.
- Further sleep characteristics are to be included in the evaluation in order to allow for a more complete conclusion. Among other things, parameters such as total time in bed, time of getting up, and sleep quality are to be incorporated into future studies, which should provide new scientific insights.
- In addition, it is planned to conduct a study with other wearable devices (which also includes smartwatches from other manufacturers) to be able to compare the measurement results. Due to a broader set of hardware devices, an extended study would permit us to determine whether a certain generalisation of the results and conclusions regarding the possibility of substituting subjective measurement with objective measurement across devices is possible.
- Performing a study with an extended set of devices additionally to wearables (e.g., smartwatch, PSG, sensors placed under the mattress) could be another direction of future research aiming to comprehensively compare different measurement approaches.

## Figures and Tables

**Table 1:** Descriptive statistics of the measured sleep characteristics for both the sleep diary and smartwatch measurements for 166 overnight recordings.

Characteristic	Mean	Median	SD	IQR
Subjective measurement with sleep diary				
Falling asleep time	00:21	00:19	00:54	01:07
Waking up time	07:27	07:29	01:03	01:24
Total Sleep Time	06:57	06:57	00:59	01:19
Sleep Efficiency	96.21%	97.79%	4.74%	3.24%
Objective measurement with smartwatch				
Falling asleep time	00:21	00:16	00:54	01:06
Waking up time	07:36	07:30	01:06	01:22
Total Sleep Time	06:33	06:33	00:56	01:12
Sleep Efficiency	89.72%	90.00%	3.43%	4.00%

**Table 2:** Statistical characteristics calculated for the absolute values of differences between the smartwatch and sleep diary measurements for 166 overnight recordings.

Characteristic	Mean	Median	SD	IQR	Sample Standard Error
Falling asleep time	00:13	00:07	00:15	00:15	00:01
Waking up time	00:12	00:06	00:12	00:17	00:00
Total Sleep Time	00:35	00:30	00:23	00:32	00:01
Sleep Efficiency	7.42%	6.77%	3.74%	5.09%	0.29%

**Table 3:** The probabilities of being within the corresponding interval for the difference between the sleep diary and smartwatch measurements.

Characteristic	Level of Similarity 1	Level of Similarity 2	Level of Similarity 3	Level of Similarity 4
*Difference interval*	*10 min*	*20 min*	*30 min*	*40 min*
Falling asleep time	58.4%	76.5%	85.5%	95.2%
Waking up time	59.0%	77.1%	88.0%	95.8%
*Difference interval*	*15 min*	*30 min*	*45 min*	*60 min*
Total Sleep Time	19.3%	51.2%	71.7%	81.9%
*Difference interval*	*5%*	*10%*	*15%*	*20%*
Sleep Efficiency	29.5%	75.3%	97.0%	100.0%

## Data Availability

The data presented in this study are available upon request from the corresponding author.
